# Effect of life cycle and venation pattern on the coordination between stomatal and vein densities of herbs

**DOI:** 10.1093/aobpla/plae007

**Published:** 2024-02-20

**Authors:** Guolan Liu, Peili Fu, Qinggong Mao, Jiangbao Xia, Wanli Zhao

**Affiliations:** Shandong Key Laboratory of Eco-Environmental Science for Yellow River Delta, Shandong University of Aeronautics, Binzhou, Shandong, China; CAS Key Laboratory of Tropical Forest Ecology, Xishuangbanna Tropical Botanical Garden, Chinese Academy of Sciences, Jinghong, Yunnan, China; Ailaoshan Station of Subtropical Forest Ecosystem Studies, Xishuangbanna Tropical Botanical Garden, Chinese Academy of Sciences, Jingdong, Yunnan, China; Key Laboratory of Vegetatcion Restoration and Management of Degraded Ecosystems, South China Botanical Garden, Chinese Academy of Sciences, Guangzhou, Guangdong, China; Shandong Key Laboratory of Eco-Environmental Science for Yellow River Delta, Shandong University of Aeronautics, Binzhou, Shandong, China; Shandong Key Laboratory of Eco-Environmental Science for Yellow River Delta, Shandong University of Aeronautics, Binzhou, Shandong, China

**Keywords:** Annual herbs, parallel venation, perennial herbs, reticulate venation, stomatal density, vein density

## Abstract

Life cycle (annual vs perennial) and leaf venation pattern (parallel and reticular) are known to be related to water use strategies in herb species and critical adaptation to certain climatic conditions. However, the effect of these two traits and how they influence the coordination between vein density (vein length per area, VLA) and stomatal density (SD) remains unclear. In this study, we examined the leaves of 53 herb species from a subtropical botanical garden in Guangdong Province, China, including herbs with different life cycles and leaf venation patterns. We assessed 21 leaf water-related functional traits for all species, including leaf area (LA), major and minor VLA, major and minor vein diameter (VD), SD and stomatal length (SL). The results showed no significant differences in mean SD and SL between either functional group (parallel venation vs reticular venation and annual vs perennial). However, parallel vein herbs and perennial herbs displayed a significantly higher mean LA and minor VD, and lower minor VLA compared to reticular vein herbs and annual herbs, respectively. There was a linear correlation between total VLA and SD in perennial and reticular vein herbs, but this kind of correlation was not found in annual and parallel vein herbs. The major VLA and minor VD were significantly affected by the interaction between life cycle and leaf venation pattern. Our findings suggested that VLA, rather than SD, may serve as a more adaptable structure regulated by herbaceous plants to support the coordination between leaf water supply and demand in the context of different life cycles and leaf venation patterns. The results of the present study provide mechanistic understandings of functional advantages of different leaf types, which may involve in species fitness in community assembly and divergent responses to climate changes.

## Teaser

We examined 21 leaf water-related functional traits of 53 herb species with different life cycles and leaf venation patterns. The results showed that there was a linear correlation between total vein length per area (VLA) and stomatal density (SD) in perennial and reticular vein herbs, but this kind of correlation was not found in annual and parallel vein herbs. The major VLA and minor vein diameter were significantly affected by the interaction between life cycle and leaf venation pattern. Our findings suggested that VLA, rather than SD, may serve as a more adaptable structure regulated by herbaceous plants to support the coordination between leaf water supply and demand in the context of different life cycles and leaf venation patterns.

## Introduction

Leaf veins serve as both mechanical support for leaf orientation towards light and conduits for the transport of nutrients, water and signalling molecules, including the xylem for water and mineral transportation and phloem for photosynthate transportation, throughout the plant. The positive correlation between vein density (vein length per area, VLA) and stomatal density (SD) has been observed in numerous species, indicating the coordination between leaf water supply and demand (Brodribb and Jordan 2011; Brodribb *et al.* 2013; Zhang *et al.* 2014, 2022; Zhao *et al.* 2016, 2023; Wen *et al.* 2020). VLA has been identified as a critical factor determining the capacity of leaf water supply in plants (Boyce 2009; Sack and Scoffoni 2013; Scoffoni *et al.* 2018), as higher photosynthetic rates require more water, resulting in increased construction costs associated with higher vein density (Brodribb *et al.* 2007; Zhao *et al.* 2020). Stomata, responsible for gas exchange between leaves and the atmosphere, play crucial roles in controlling the maximum transpiration rate and, consequently, the leaf water demand (Hetherington and Woodward 2003; Franks and Beerling, 2009). The positive linear correlation between SD and VLA allows leaves to optimize photosynthetic advantages while minimizing costs (Brodribb and Jordan 2011; Carins Murphy *et al.* 2012).

However, certain species, such as 20 terrestrial and epiphytic *Cymbidium* species, exhibit distinct water balance strategies, as they do not show a significant positive correlation between vein density and SD (Zhang *et al.* 2015). Moreover, in arid conditions, some species demonstrate an apparent over-investment in leaf venation to compensate for the negative impact of thicker leaves on photosynthesis (de Boer *et al.* 2016). Additionally, Zhao *et al.* (2016) discovered a weak coordination between vein and stomatal densities in 105 angiosperm tree species across altitudinal gradients in Southwest China. Nevertheless, there is still limited understanding of the relationship between VLA and SD in herbaceous plants with different life cycles and types of leaf veins.

The herbaceous layer plays a vital role in ecosystems, serving multiple ecological functions such as increasing diversity, preventing soil and water loss, enhancing soil nutrition, amending soil structure, promoting seedling growth, improving microclimate and facilitating ecological restoration (Drake *et al.* 2019; Friedman 2020). Herbaceous plants have either an annual or perennial life cycle; annuals are plants that germinate, grow, bloom and die within a year, whereas perennials have a lifespan of more than 2 years (Gonzalez-Paleo and Ravetta 2018; Nagahama and Yahara 2019). These two groups are believed to employ different strategies for carbon gain and water use (Garnier and Laurent 1994; Muir 2018). For instance, in the oasis-desert transition zone, annual herbs were found to have significantly larger leaf area (LA) and higher total nitrogen content compared to perennial herbs, while perennial herbs exhibited significantly higher leaf dry matter and carbon content than annual herbs (Wang *et al.* 2022). Another study on the Loess Plateau revealed that annual herbs generally had higher SD but smaller stomatal size on each side of the leaf epidermis compared to perennial herbs, although there was no significant difference in stomatal relative area (Sun *et al.* 2021). However, the differences in leaf vein traits between annual and perennial herbaceous plants, as well as the variations in the relationship between leaf stomatal and vein traits, remain unclear. Based on previous research findings (Brodribb and Jordan 2011; Sun *et al.* 2021), we hypothesize that annual herbs would exhibit higher VLA than perennial herbs and that a positive linear correlation between VLA and SD would exist in herbaceous plants with different life cycles, as it optimizes photosynthetic yield.

**Figure 1.**
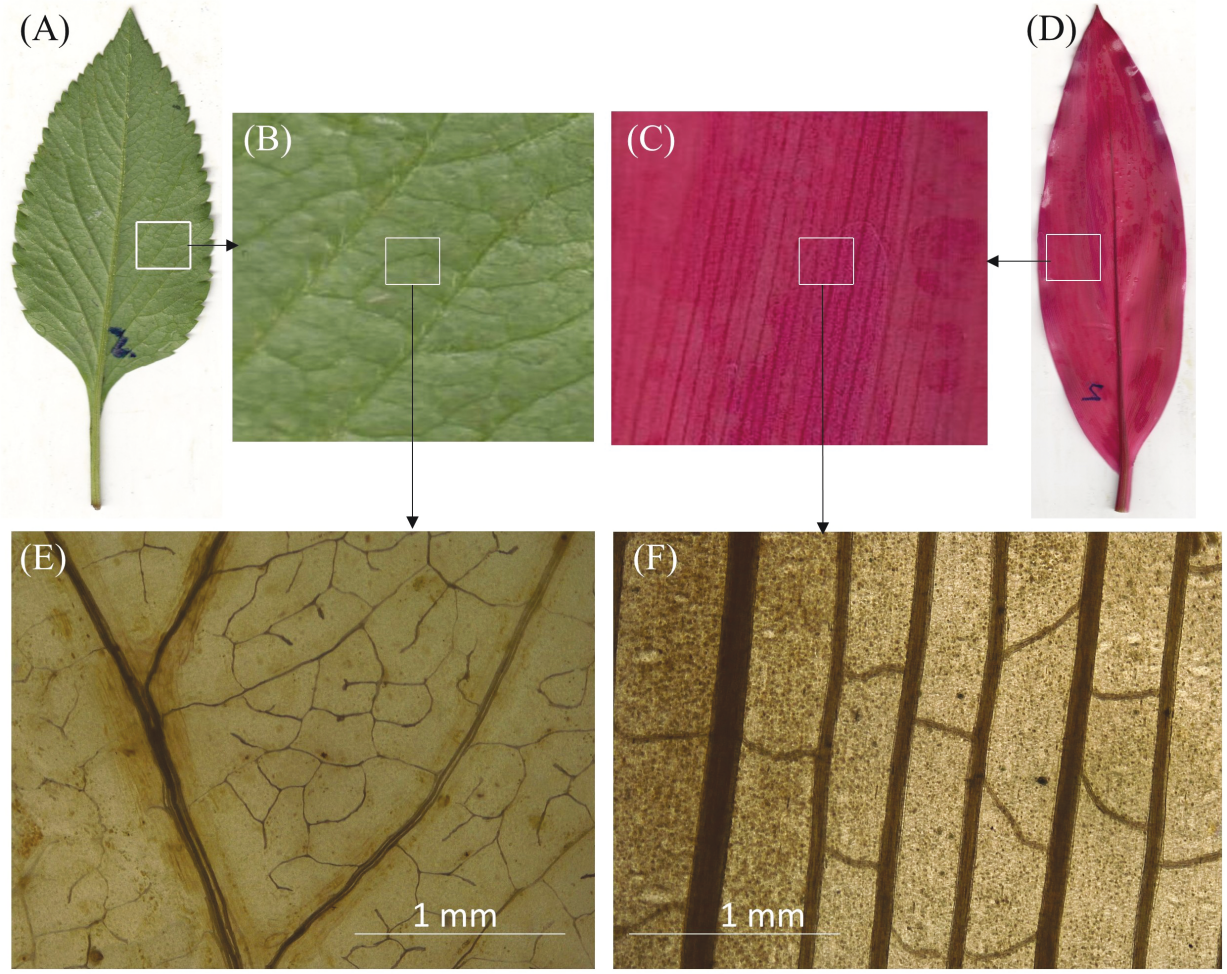
The leaves and veins of annual and perennial herb plants. (a) *Bidens Pilosa*, (b) *Cordyline fruticose*, (e) reticulate venation, (f) parallel venation.

Herbaceous plants can also be classified into two groups based on their leaf venation patterns: parallel vein herbs and reticular vein herbs (Fig. 1). These two groups exhibit distinct morphological differences, making them easily distinguishable (Przybylska *et al.* 2020). Parallel vein herbs are typically monocotyledonous, while reticular vein herbs are usually dicotyledonous (Sack and Scoffoni 2013). In monocotyledonous herbaceous plants, major veins dominate the leaf vein network, whereas, in dicotyledonous herbaceous plants, minor veins constitute over 80 % of the leaf veins (Sack and Scoffoni 2013; Zhao *et al.* 2020; Robil *et al.* 2021). These major and minor veins serve different functions in water transport (Sack *et al.* 2008). In optimized irrigation systems, major veins act as high-capacity lateral-supply ‘mainlines’, with the leaf hydraulic conductance determined by the total number and size of xylem conduits, regardless of major vein density (Sack and Frole 2006; Sack *et al.* 2008). Conversely, the minor vein system operates as a ‘distribution network’, where increased vein density enhances conductance by providing a larger surface area for water transfer to the mesophyll (Sack and Holbrook 2006). However, the variations in the relationship between leaf vein and stomatal vein traits in monocotyledonous and dicotyledonous herbaceous plants remain unclear. We hypothesize that, to optimize photosynthetic yield, a coordination between VLA and SD would exist in herbaceous plants with different leaf venation patterns.

A comparison of the coordination between the leaf water supply and demand of different species under the same environmental conditions, or the same species acclimated to different environmental conditions, could indicate different adaptation strategies (Carins Murphy *et al*. 2013; Zhao *et al*. 2016, 2020). The slope and intercept of linear regression analyses between stomatal and minor vein densities were used for comparison (Carins Murphy *et al.* 2013; Zhang *et al.* 2022). For example, the correlation between stomatal and minor vein density in *Toona ciliata*, as observed by Carins Murphy *et al.* (2012, 2013), remained consistent in the leaves produced by plants acclimated to different vapour pressures or irradiance treatments. The stomatal number per minor vein length (SV) has also been used to compare the difference in the coordination between leaf water supply and demand. For example, Zhao *et al*. (2017) found that three leguminous species under same light conditions exhibited a stable SV, indicating the coordination between leaf water supply and demand, and when environmental conditions changed, SV also changed accordingly. Additionally, Zhang *et al.* (2022) found a consistent SV across different growth forms (trees, shrubs and herbs) comprising 194 species. Furthermore, the investigation of SV as a novel functional trait in different species, particularly in herbs with diverse life cycles and leaf venation patterns, would provide valuable insights into plant biology and enhance our understanding of the coordination between leaf water supply and demand.

Indeed, considering only one aspect, either the life cycle or leaf vein pattern, would overlook the crucial interaction between these two factors (Wang *et al.* 2022). The life cycle and leaf venation pattern are interconnected because they are both influenced by genetic and environmental factors, and they can have reciprocal effects on each other, which reflects the adaptation of plants to optimize resource acquisition and allocation strategies for their respective life strategies. Therefore, examining both aspects independently and in combination is necessary to fully comprehend the complex relationship and interactions between the life cycle and leaf vein pattern in herbaceous plants and their implications for water use strategies. In this study, we separated herbs according to their life cycle and leaf venation pattern and divided them into four groups: annual dicotyledonous herbs (ADH), annual monocotyledonous herbs (AMH), perennial dicotyledonous herbs (PDH) and perennial monocotyledonous herbs (PMH). We then conducted an assessment of 21 leaf water-related functional traits for 53 herbaceous species from a subtropical botanical garden in Guangdong Province, China. The traits included LA, major and minor VLA, major and minor vein diameter (VD), theoretical maximum stomatal conductance, SD, SV and stomatal length (SL).

## Material and methods

### Site and sampling

This study was conducted at the SCBG (23°10ʹ N, 113°21ʹ E, elevation 41 m), Chinese Academy of Sciences, Guangzhou City, Guangdong Province, China. The mean annual temperature in the garden is 21.7 °C and the mean annual precipitation is 1761 mm (with more than 80 % rain from May to September). In July 2017, we collected 4–6 mature leaves from various individuals of 53 herbaceous species exposed to full sunlight. To ensure their preservation for future use, the collected leaves were stored in a refrigerator at 4 °C. The species collected, included 12 ADH, 7 AMH, 14 PDH and 20 PMH. The species list is provided in Supporting Information—Supplementary Data 1.

The collected leaves were scanned (HP Scanjet G3110; Hewlett-Packard Development Co., Palo Alto, CA, USA) to obtain leaf images. Then, we used Image J software (http://rsbweb.nih.gov/ij/index.html) to measure LA. Leaf samples were stored in 70 % alcohol until further analyses.

### SD and modelled maximum stomatal conductance

The SD, SL and stomatal width (SW) were determined from the paraxial and abaxial cuticles of the leaves. We applied a clear nail varnish to a 1 cm^2^ patch on the middle part of the leaf surface. After 3 min, the nail polish was removed, mounted on a glass slide and observed under a microscope (LEICA DM 2500, Germany). Stomatal images were taken at ×200 and ×400 magnification. For each species, approximately 20–30 pictures were taken, and SL and width were measured in more than 20 stomata.

For each species, we also estimated the theoretically modelled maximum stomatal conductance (*g*_max_), as reported by Franks and Farquhar (2001):


$$\begin{array}{l} g_{max}=\frac{d}{v}\times D\times \frac{a}{l+\pi /2\sqrt{a/\pi }}, \end{array}$$ (1)

where *d* is the diffusivity of water in air (24.9 × 10^−6^ m^2^ s^−1^, 25 °C); *υ* is the molar volume of air (24.4 × 10^−3^ m^3^ mol^−1^, 25 °C, 101.3 kPa), *D* is the SD, *a* is the maximum pore area and *l* is the pore depth that is represented by mean SW; the maximum pore area was calculated from the SL (Brodribb *et al*. 2013).

### Leaf vein measurements and categories

Leaves that were used to measure stomatal traits were also used to measure VLA. ImageJ was used to measure the VLA for the different vein categories. The leaves were placed in bottles containing a 5 % NaOH aqueous solution and heated in a water bath (Yiheng HWS24, Shanghai, China) until the veins were exposed. The leaves were soaked in distilled water for 30 min, stained with a 1 % methylene blue solution, rinsed again, mounted on slides and photographed. We measured the major VLA (1–3°) separately, but for the minor VLA, the 4° and higher orders were grouped into one class. The major VDs (major VD) were measured for different orders from the middle of the leaves, and the mean minor VD (minor VD) was calculated for orders 4°. The number of stomata per vein length (no. mm^−1^) was calculated by dividing SD by total VLA.

We estimated the xylem construction cost of the leaf veins using a dimensionless index of cell wall volume per LA (CC; McKown *et al.* 2010). A modified yet simplified method of Schneider *et al.* (2017) for lumen diameter and conduit density per vein order determination was applied for total VD determination based on the assumption that both variables correlate with VD. Thus, we used the following equation to calculate the xylem construction cost of leaf veins:


$$\begin{array}{l} CC=\;\;\;\underset{i\;\;\;=\;\;\;1}{\overset{v}{\sum }}\;\pi \times d_{i}\times D_{i} \end{array}$$ (2)

where *d*_i_ is the diameter of vein order *i* and *D*_i_ is the density of the same order.

### Data analyses

One-way analysis of variance (ANOVA) was used to compare the differences in mean species values of leaf functional traits between annual and perennial herbs and between dicotyledon and monocotyledon herbs. Two-way ANOVA was used to analyse the effects of life cycle, leaf venation pattern and their interactions on functional traits. Principal component analysis (PCA) was used to analyse the correlations between plant functional traits and the distributions of the 53 species along the PCA axes. The raw data for the functional traits were square root-transformed before analysis to meet the normality assumption. Statistical analyses were conducted using SPSS software (version 16.0; SPSS Inc.). The bivariate trait relationships were analysed with Pearson’s correlation and the differences in the slope or intercept of bivariate relationships between different life cycle and leaf venation pattern were examined with standardized major axis tests using SMATR (v2.0) (Warton *et al*. 2006).

## Results

There were no significant differences in SD, SL, maximum modelled *g*_max_, 1° VLA, SV or minor CC between dicotyledonous and monocotyledonous herbs or between annual and perennial herbs (Table 1). Monocotyledonous and perennial herbs had significantly lower mean minor VLA but higher LA than dicotyledonous and annual herbs (Table 1). PMH had the lowest minor VLA, and ADH had the highest minor VLA (Table 2). We found that minor VLA were significantly affected by both the life cycle and leaf venation pattern (Table 3). We also found that 3° VLA, Major VLA, 2° VD, 3° VD and minor VD were significantly affected by the interaction between life cycle and leaf venation pattern (Table 3).

**Table 1.** Leaf functional traits (mean ± standard error) of 53 angiosperm herbs with different leaf venation patterns and life cycles.

Traits	Units	Leaf venation pattern		Life cycle	
		DH (26)	MH (27)	AH (19)	PH (34)
LA	cm^2^	23.8 ± 3.9	59.9 ± 7.6^***^	27.5 ± 4.3	50.4 ± 7^*^
SD	no. mm^−2^	339 ± 31.5	390 ± 61.3^ns^	391 ± 46.6	350 ± 47.5^ns^
SL	μm	24.9 ± 1.0	25.1 ± 1.44^ns^	23 ± 1.2	26.1 ± 1.1^ns^
*g*_max_	μmol H_2_O m^−2^s^−1^	0.68 ± 0.04	0.76 ± 0.08^ns^	0.73 ± 0.06	0.71 ± 0.07^ns^
1°VLA	mm mm^−2^	0.07 ± 0.02	0.11 ± 0.03^ns^	0.06 ± 0.01	0.11 ± 0.03^ns^
2°VLA	mm mm^−2^	0.22 ± 0.02	0.73 ± 0.1^***^	0.43 ± 0.1	0.51 ± 0.08^ns^
3°VLA	mm mm^−2^	0.46 ± 0.05	4.4 ± 0.7^***^	2.72 ± 0.98	2.3 ± 0.46^ns^
Major VLA	mm mm^−2^	0.76 ± 0.07	5.2 ± 0.8^***^	3.2 ± 1.06	2.91 ± 0.52^ns^
Minor VLA	mm mm^−2^	6.4 ± 0.3	2.85 ± 0.3^***^	6.8 ± 0.4	4.8 ± 0.4^**^
Total VLA	mm mm^−2^	7.15 ± 0.3	6.05 ± 0.7^ns^	7.9 ± 0.7	5.9 ± 0.4^**^
1°VD	μm	547 ± 53	648 ± 94^ns^	501 ± 49	653 ± 79^ns^
2°VD	μm	114 ± 12.1	110 ± 13.7^ns^	108 ± 11.7	114 ± 12.5^ns^
3°VD	μm	27.6 ± 1.4	39.1 ± 2.9^***^	30.7 ± 2	35 ± 2.6^ns^
Minor VD	μm	8.3 ± 0.3	19.7 ± 1.4^***^	7.6 ± 0.3	13.1 ± 1.2^**^
1°CC	–	0.1 ± 0.01	0.1 ± 0.01^ns^	0.08 ± 0.01	0.11 ± 0.01^ns^
2°CC	–	0.07 ± 0.01	0.19 ± 0.02^***^	0.11 ± 0.02	0.15 ± 0.02^ns^
3°CC	–	0.04 ± 0.0	0.41 ± 0.05^***^	0.22 ± 0.07	0.23 ± 0.04^ns^
Major CC	–	0.21 ± 0.02	0.7 ± 0.06^***^	0.41 ± 0.09	0.49 ± 0.05^ns^
Minor CC	–	0.16 ± 0.0	0.18 ± 0.03^ns^	0.16 ± 0.01	0.17 ± 0.01^ns^
Total CC	–	0.37 ± 0.02	0.75 ± 0.05^***^	0.52 ± 0.07	0.59 ± 0.04^ns^
SV	no. mm^−1^	52.4 ± 3.6	73 ± 10.5^ns^	56.8 ± 9.2	66.3 ± 7.4^ns^

LA, leaf area; SD, stomatal density; SL, stomatal length, *g*_max_, maximum modelled stomatal conductance; VLA, vein length per area; VD, vein diameter; SV, stomatal number per vein length; CC, construction cost of minor vein network per leaf area. AH, annual herbs; DH, dicotyledonous herbs; MH, monocotyledonous herbs; PH, perennial herbs. Significant differences between annual and perennial herbs, and between monocotyledonous and dicotyledonous herbs according to Mann–Whitney *U* test.

^*^ *P* < 0.05;

^**^ *P* < 0.01;

^***^ *P* < 0.001; ns, *P* > 0.05.

**Table 2.** Leaf functional traits for annual and perennial herbs and for monocotyledonous and dicotyledonous herbs.

Traits	Units	Annual herbs		Perennial herbs	
		ADH (12)	AMH (7)	PDH (14)	PMH (20)
LA	cm^2^	23.8 ± 4.9^a^	34 ± 8^a^	23.8 ± 5.8^a^	69 ± 9.2^b^
SD	no. mm^−2^	414 ± 50^a^	352 ± 98^ab^	276 ± 30.7^b^	403 ± 77^ab^
SL	μm	22.4 ± 1.1^a^	24.2 ± 2.9^ab^	27 ± 1.4^b^	25.4 ± 1.7^ab^
*g*_max_	μmol H_2_O m^−2^s^−1^	0.79 ± 0.06^a^	0.64 ± 0.12^ab^	0.58 ± 0.04^b^	0.8 ± 0.11^ab^
1°VLA	mm mm^−2^	0.05 ± 0.01^a^	0.09 ± 0.01^b^	0.09 ± 0.03^b^	0.12 ± 0.04^b^
2°VLA	mm mm^−2^	0.18 ± 0.02^a^	0.85 ± 0.18^b^	0.26 ± 0.04^a^	0.68 ± 0.12^b^
3°VLA	mm mm^−2^	0.36 ± 0.05^a^	6.76 ± 1.85d	0.55 ± 0.07^b^	3.52 ± 0.66c
Major VLA	mm mm^−2^	0.58 ± 0.06^a^	7.7 ± 1.97c	0.91 ± 0.11^b^	4.32 ± 0.73c
Minor VLA	mm mm^−2^	7.1 ± 0.4^a^	4	5.8 ± 0.3^b^	2.7 ± 0.4c
Total VLA	mm mm^−2^	7.7 ± 0.4^a^	8.3 ± 1.7^a^	6.7 ± 0.4^ab^	5.3 ± 0.6^b^
1°VD	μm	546 ± 64^a^	424 ± 72^a^	547 ± 82^a^	727 ± 120^a^
2°VD	μm	129 ± 14.5^a^	71 ± 9.6^b^	101 ± 17.9^b^	124 ± 17.3^b^
3°VD	μm	31.8 ± 2.2^a^	28.9 ± 3.9^ab^	24.1 ± 1.1^b^	42.7 ± 3.4c
Minor VD	μm	7.6 ± 0.4^a^	7.9	9 ± 0.2^b^	21.3 ± 1.3c
1°CC	–	0.07 ± 0.01^a^	0.1 ± 0.01^b^	0.12 ± 0.02^b^	0.1 ± 0.01^b^
2°CC	–	0.07 ± 0.01^a^	0.18 ± 0.03^b^	0.08 ± 0.01^a^	0.19 ± 0.03^b^
3°CC	–	0.03 ± 0^a^	0.53 ± 0.13^b^	0.04 ± 0.01^a^	0.37 ± 0.05^b^
Major CC	–	0.18 ± 0.02^a^	0.81 ± 0.15^b^	0.23 ± 0.03^a^	0.66 ± 0.06^b^
Minor CC	–	0.16 ± 0.01^a^	0.1	0.16 ± 0.01^a^	0.19 ± 0.03^a^
Total CC	–	0.34 ± 0.01^a^	0.82 ± 0.14^b^	0.4 ± 0.04^a^	0.73 ± 0.05^b^
SV	no. mm^−1^	57.8 ± 5^ab^	55 ± 24.6^ab^	47.8 ± 4.9^a^	79.3 ± 11.4^b^

LA, leaf area; SD, stomatal density; SL, stomatal length; *g*_max_, maximum modelled stomatal conductance; VLA, vein length per area; VD, vein diameter; SV, stomatal number per vein length; CC, construction cost of minor vein network per leaf area. ADH, annual dicotyledonous herbs; AMH, annual monocotyledonous herbs; PDH, perennial dicotyledonous herbs; PMH, perennial monocotyledonous herbs. Different letters indicate significant differences according to Kruskal–Wallis rank sum test (*P* < 0.05).

**Table 3.** Impact of life cycle and leaf venation pattern on leaf traits in 53 angiosperm herbs.

Traits	Life cycle		Leaf venation pattern		Life cycle × leaf venation pattern	
	*F*	*P*	*F*	*P*	*F*	*P*
LA	0.10	ns	2.50	ns	3.95	ns
SD	0.21	ns	2	ns	1.61	ns
SL	3.05	ns	0.01	ns	0.81	ns
*g*_max_	0.02	ns	0.03	ns	3.50	ns
1° VLA	18.80	ns	13.99	ns	0.05	ns
2° VLA	0.12	ns	20.23	ns	1.19	ns
3° VLA	0.78	ns	7.41	ns	5.34	^*^
Major VLA	0.68	ns	8.10	ns	5.23	^*^
Minor VLA	1164	^*^	6568	^**^	0.002	ns
Total VLA	3.64	ns	0.17	ns	1.95	ns
1° VD	1.02	ns	0.04	ns	1.73	ns
2° VD	0.09	ns	0.19	ns	4.59	^*^
3° VD	0.08	ns	0.54	ns	10.8	^**^
Minor VD	1.50	ns	1.11	ns	13.7	^***^
1° CC	0.95	ns	0.17	ns	1.68	ns
2° CC	2.77	ns	237.1	^*^	0.08	ns
3° CC	0.85	ns	23.38	ns	2.79	ns
Major CC	0.18	ns	27.54	ns	2.15	ns
Minor CC	0.91	ns	0.14	ns	1.53	ns
Total CC	0.06	ns	30.6	ns	1.50	ns
SV	0.17	ns	0.70	ns	2.02	ns

LA, leaf area; SD, stomatal density; SL, stomatal length; *g*_max_, maximum modelled stomatal conductance; VLA, vein length per area; VD, vein diameter; SV, stomatal number per vein length, CC, construction cost of minor vein network per leaf area.

^*^ *P* < 0.05;

^**^ *P* < 0.01;

^***^ *P* < 0.001; ns, *P* > 0.05.

**Figure 2.**
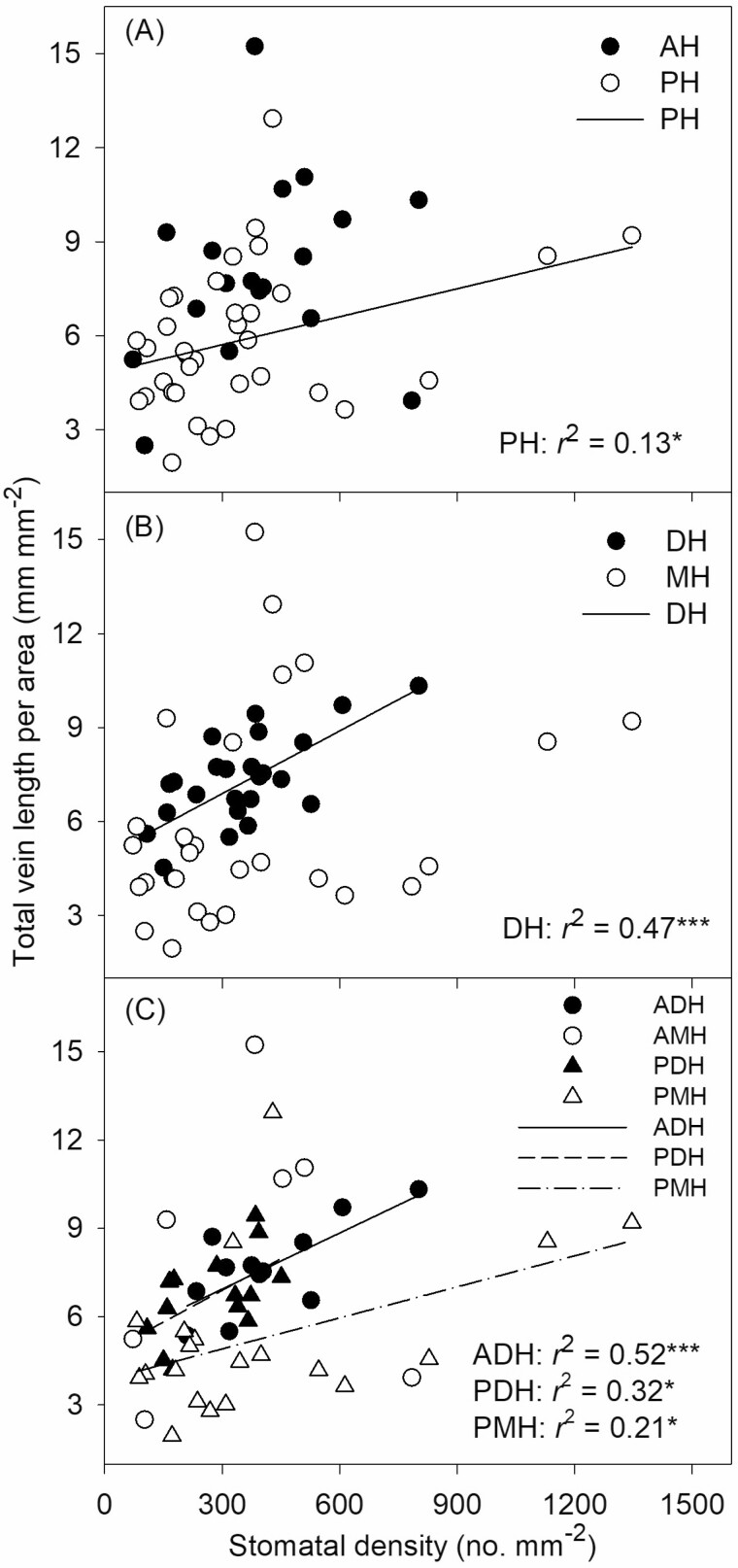
Correlations between SD and total vein length per area of angiosperm herbs with different life cycle and leaf venation patterns. Each symbol represents one species. *P > 0.05; **P < 0.01; ***P < 0.001. AH, annual herbs; PH, perennial herbs; DH, dicotyledonous herbs with reticulate venation; MH monocotyledonous herbs with parallel venation; ADH, annual dicotyledonous herbs; AMH, annual monocotyledonous herbs; PDH,perennial dicotyledonous herbs; PMH, perennial monocotyledonous herbs.

A significant positive correlation was found between total VLA and SD in perennial herbs (*r*^2^ = 0.13, *P* < 0.05) but not in annual herbs (Fig. 2A). Similarly, significant correlations were found between total VLA and SD in dicotyledonous herbs (*r*^2^ = 0.47, *P* < 0.001) but not in monocotyledonous herbs (Fig. 2B). When the 53 herbs were separated into 4 groups according to their life cycle and leaf venation pattern, we found a significant positive correlation between total VLA and SD in ADH (*r*^2^ = 0.52, *P* < 0.001), PDH (*r*^2^ = 0.32, *P* < 0.05), and PMH (*r*^2^ = 0.21, *P* < 0.05) but not in annual monocotyledonous herbs (Fig. 2C).

**Figure 3.**
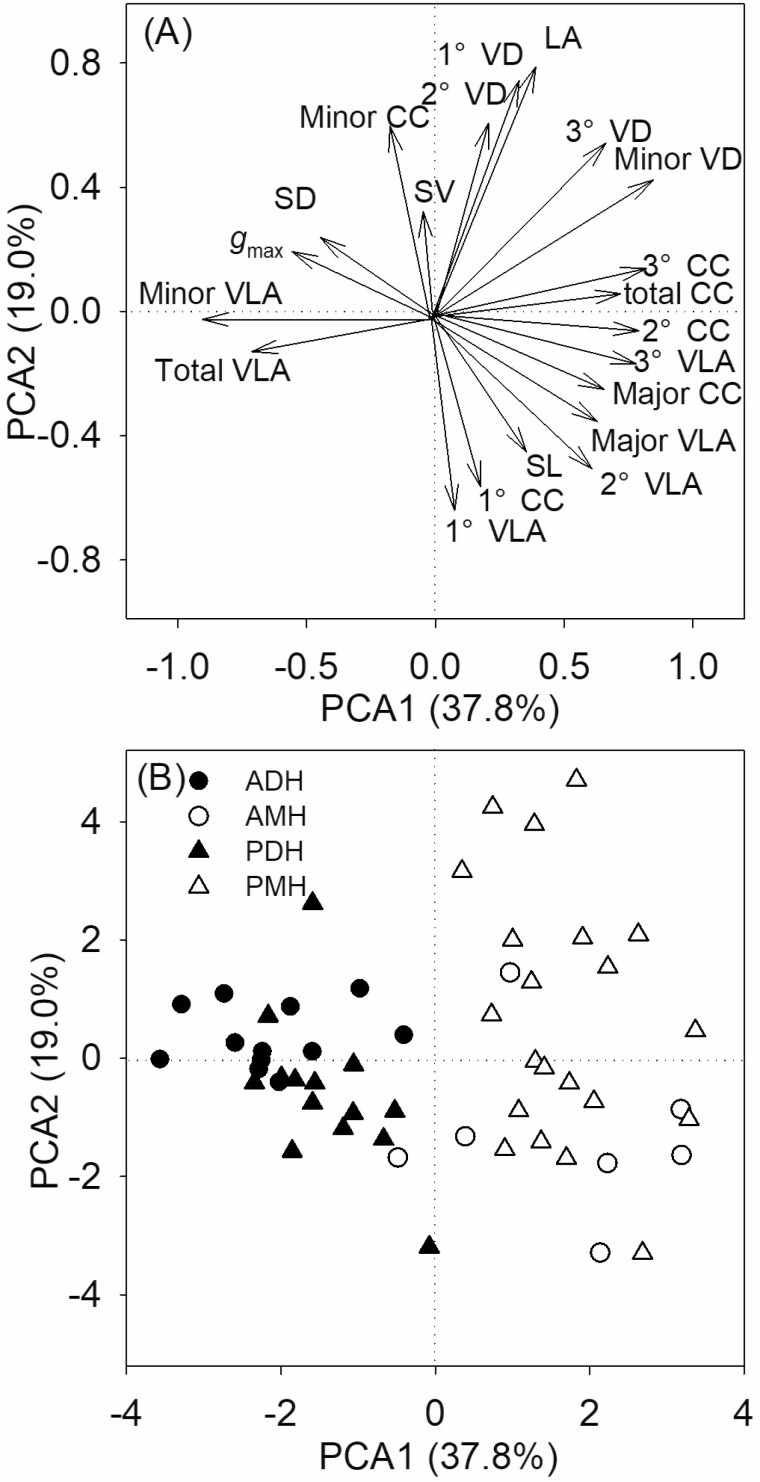
First two axes of the PCA for the leaf functional traits and loading of the 53 species along the first 2 PC axes. The trait codes are as in Table 1. ADH, annual dicotyledonous herbs; AMH, annual monocotyledonous herbs; PDH, perennial dicotyledonous herbs; PMH, perennial monocotyledonous herbs.

The first axis of the PCA (PCA1) accounted for 37.8 % of the variance and the second axis (PCA2) explained 19.0 % of the variance among the 21 variables (Fig. 3A). PCA1 was loaded with minor VD, total CC and major VLA on the positive side and with *g*_max_, SD and total VLA on the negative side, whereas PCA2 was loaded with LA on the positive side (Fig. 3A). Dicotyledonous and monocotyledonous herbs were separated from each other along PCA2, with monocotyledonous herbs distributed on the positive side and dicotyledonous herbs on the negative side of PCA1 (Fig. 3B).

**Figure 4:**
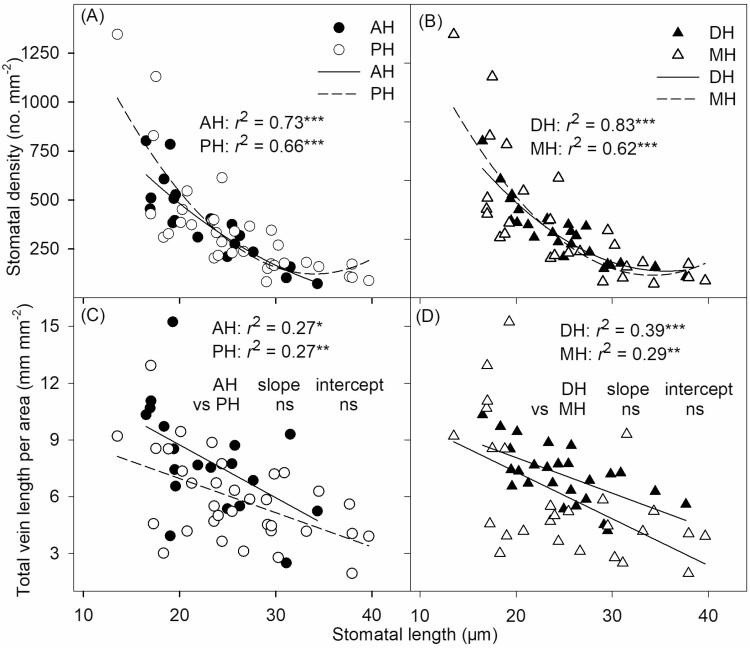
Correlation of SL with stomatal density (SD) and total vein length per area of angiosperm herbs with different life cycle (A and C) and leaf venation patterns (B and D). Each symbol represents one species, and relationships were significant for each group. AH, annual herbs; PH, perennial herbs; DH, dicotyledonous herbs with reticulate venation; MH, monocotyledonous herbs with parallel venation. *P < 0.05; **P < 0.01; ***P < 0.001; ns, *P* > 0.05.

A significant negative correlation was found between SD and SL in annual herbs (*r*^2^ = 0.73, *P* < 0.001), perennial herbs (*r*^2^ = 0.66, *P* < 0.001), dicotyledonous herbs (*r*^2^ = 0.83, *P* < 0.001) and monocotyledonous herbs (*r*^2^ = 0.62, *P* < 0.001; Fig. 4A and B). Similarly, significant correlations were found between total VLA and SL in annual herbs (*r*^2^ = 0.27, *P* < 0.05), perennial herbs (*r*^2^ = 0.27, *P* < 0.01), dicotyledonous herbs (*r*^2^ = 0.39, *P* < 0.001) and monocotyledonous herbs (*r*^2^ = 0.29, *P* < 0.01; Fig. 4C and D), and the regression slopes and intercepts for AH and DH were not significantly different from those for PH and MH (Fig. 4C and D).

## Discussion

An important finding of this study was that both the life cycle and leaf venation pattern had significant effects on VLA and VD, but not on SD and SL. Under the same homogenous garden environment, herbs may respond to similar environmental factors by using different water supply strategies to meet the same transpiration demand. In previous studies, SD and VLA have shown different or even opposite trends in response to the same environmental factors (Uhl and Mosbrugger 1999; Hu *et al*. 2014). As life cycle and leaf venation patterns have different effects on VLA and SD, coordination between them was not found in annual herbs, monocotyledonous herbs, or annual monocotyledonous herbs in this study.

To maximize photosynthetic yield, plants should maintain a balance between hydraulic supply and transpirational loss (Brodribb and Jordan 2011; Sun *et al*. 2014). The correlation between SD and VLA has been demonstrated in other studies across different species, genera and families (Carins Murphy *et al*. 2012; Brodribb *et al*. 2013; Zhang *et al*. 2014). Our study further revealed the effect of the life cycle and leaf venation pattern on this coordination. Other studies have also found no correlation between SD and VLA in many species (Zhang *et al*. 2015, 2022; Zhao *et al*. 2016), which suggests that the relationship between these two traits might vary among different plant species. Zhang *et al*. (2022) found that the positive SD-VLA relationship only in shrubs and herbs, but not in trees on the northern slope of Taibai Mountain in China. Zhao *et al*. (2016) found no correlation between SD and vein density in Fagaceae and Lauraceae. In the present study, the lack of this relationship in annual herbs may have been due to the presence of a few outlier species (recall Fig. 2). One species, *Oryza sativa* had a high SD (784.0 mm^–2^) but a low total VLA (3.9 mm^–2^); in contrast, *Digitaria sanguinalis* had a high total VLA (15.2) but a relatively low SD (383.0 mm^–2^). This likely reflects the different paths of adaptation among plant taxa (Woodward 1987; Bresson *et al*. 2011). After removing these two species, the remaining annual herbs showed a significant positive correlation between SD and total VLA (*r*^2^ = 0.44, *P* < 0.05).

Maintaining an appropriate water balance in leaves relies on the coordinated interplay between leaf veins and stomata, which is crucial for regulating water supply and demand (Carins Murphy *et al.* 2016; Brodribb *et al.* 2017; Zhang *et al.* 2022). Our findings demonstrate that both the life cycle and leaf venation pattern of herbaceous plants significantly influences the equilibrium between leaf water supply and demand, as indicated by the relationship between SD and total VLA (recall Fig. 2). However, it is important to note that the absence of a correlated SD and total VLA in annual herbs and monocotyledonous herbs could be attributed to a few exceptional species. Interestingly, SV showed no significant difference between dicotyledonous and monocotyledonous herbs or between annual and perennial herbs (Table 1), suggesting a consistent water supply and demand balance relationship. SV, as a functional parameter, is less influenced by individual outliers and effectively represents the balance between water supply and demand in leaves, potentially due to its ability to mitigate the influence of LA (Zhao *et al.* 2017). Consequently, SV offers a promising approach for investigating the relationship between SD and VLA across different plant groups.

The annual and perennial life cycles are two different adaptations to environmental conditions that subsequently cause differences in functional traits between the two classes of herb species (Friedman 2020). Many functional traits would differ between annual and perennial herbs (Garnier and Laurent 1994). For example, annual herbs have leaves with higher gas exchange and photosynthetic rates than those of perennial herbs (Gonzalez-Paleo and Ravetta 2018). In our study, we also observed that annual herbs possessed a greater number of smaller stomata compared to perennial herbs, although no significant differences in stomatal traits were found between these two types of herbs (Table 1). Previous research has revealed that annual plants employ random survival strategies, germinating in response to rainfall (Volis *et al.* 2002), while short-lived plants complete their life cycles within a brief growth period. Perennial plants enhance stress resistance through the regulation of growth rates and physiological adjustments (Aranda *et al.* 2001; Heilmeier *et al.* 2002). Smaller stomata provide an advantage for annual plants, enabling them to quickly respond to environmental changes by promptly opening and closing stomata, thereby regulating moisture exchange (Muir 2018; Sun *et al.* 2021). In contrast, perennial plants have the ability to persist in a specific environment for an extended period and exhibit morphological adaptations that optimize survival (Sun *et al.* 2021). Consequently, in comparison to annual herbs, perennial herbs do not require rapid stomatal closure to minimize water loss.

The effects of the life cycle and leaf venation pattern on the leaf traits were similar in this study. By performing two-way ANOVA, we found that the life cycle only had an effect on minor VLA, and leaf venation pattern had an effect on minor VLA and 2° CC (Table 3). The life cycle is less effective than the leaf venation pattern in distinguishing herbs because herbs can be separated PCA2 into dicotyledonous and monocotyledonous herbs. The life cycle can be changed by the environment, and perennial herbs under cold climatic conditions might change to annual herbs (Friedman 2020). However, the leaf venation pattern is a consequence of long-term evolution. In most studies, herbs are classified according to their life cycle or leaf venation pattern. It is very rare to classify herb species into four categories according to the life cycle and leaf venation pattern; in particular, the differences in functional traits and distribution patterns of various groups require further study.

## Conclusions

In conclusion, we found a linear correlation between total VLA and SD in perennial and reticular vein herbs, but this kind of correlation was not found in annual and parallel vein herbs. The major VLA and minor VD were significantly affected by the interaction between life cycle and leaf venation pattern. Our findings suggested that VLA, rather than SD, may serve as a more adaptable structure regulated by herbaceous plants to support the coordination between leaf water supply and demand in the context of different life cycles and leaf venation patterns. The results of the present study provide mechanistic understandings of the functional advantages of different leaf types, which may be involved in determining species fitness in community assembly and divergent responses to climate changes.

## Supplementary Material

plae007_suppl_Supplementary_Data
